# PtdIns 3-Kinase Orchestrates Autophagosome Formation in Yeast

**DOI:** 10.1155/2011/498768

**Published:** 2011-01-04

**Authors:** Keisuke Obara, Yoshinori Ohsumi

**Affiliations:** ^1^Faculty of Pharmaceutical Sciences, Hokkaido University, Kita-12 jo Nishi-6 chome, Kitaku, Sapporo 060-0812, Japan; ^2^Integrated Research Institute, Tokyo Institute of Technology, 4259-S2-12 Nagatsuda-cho, Midoriku, Yokohama 226-8503, Japan

## Abstract

Eukaryotic cells can massively transport their own cytoplasmic contents into a lytic compartment, the vacuole/lysosome, for recycling through a conserved system called autophagy. The key process in autophagy is the sequestration of cytoplasmic contents within a double-membrane structure, the autophagosome. Autophagosome formation requires the elaborate cooperation of Atg (*a*u*t*opha*g*y-related) proteins and lipid molecules. Phosphorylation of phosphatidylinositol (PtdIns) by a PtdIns 3-kinase, Vps34, is a key step in coordinating Atg proteins and lipid molecules. Vps34 forms two distinct protein complexes, only one of which is involved in generating autophagic membranes. Upon induction of autophagy, PtdIns(3)*P*, the enzymatic product of PtdIns 3-kinase, is massively transported into the lumen of the vacuole *via* autophagy. PtdIns(3)*P* is enriched on the inner membrane of the autophagosome. PtdIns(3)*P* recruits the Atg18−Atg2 complex and presumably other Atg proteins to autophagic membranes, thereby coordinating lipid molecules and Atg proteins.

## 1. Membrane Dynamics of Autophagy

**Figure 1:**
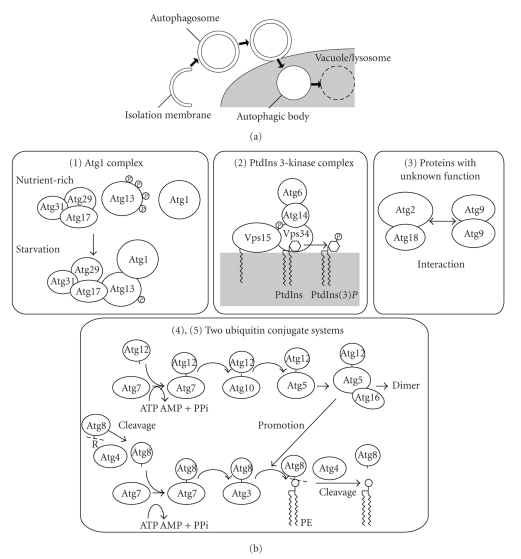
Membrane dynamics of autophagy and 5 groups of Atg proteins that are essential for autophagosome formation. (a) A schematic diagram of the membrane dynamics during autophagy. Upon autophagy induction, the isolation membrane elongates to enclose a portion of the cytoplasm, and the ends of the isolation membrane fuse to generate a closed double-membrane structure, the autophagosome. The outer membrane of the autophagosome fuses with the vacuole/lysosome to release the inner membrane structure, the autophagic body, into the lumen where the autophagic body and the enclosed cargo are degraded for recycling. (b) Five groups of Atg proteins are essential for autophagosome formation. Currently, 18 Atg proteins are thought to be essential for autophagosome formation. Based on their functions, they are classified into 5 groups. Reproduced with modification from [8].

Eukaryotic cells are equipped with a self-digesting system called macroautophagy (hereafter, autophagy). Using this system, cells can degrade a portion of their cytoplasmic contents, occasionally including organelles, within a lytic compartment called the vacuole or lysosome. Autophagy is considered a primarily cell survival mechanism that enables macromolecules to be recycled under nutrient-limited conditions. However, recent studies have shown that autophagy is a versatile system that is also involved in the clearance of protein aggregate precursors, defense against invading pathogens, cell differentiation, and so forth [1–4]. Autophagy involves unique membrane dynamics, which has been predominantly shown through detailed electron microscopic analyses of starved yeast cells [5, 6]. Upon the induction of autophagy, a cup-shaped isolation membrane emerges in the cytoplasm and elongates to enclose a portion of the cytoplasm. The isolation membrane fuses at its ends to become a closed double-membrane structure called the autophagosome. The autophagosome then fuses with a vacuole or lysosome, where the inner membrane structure is released into the lumen as the autophagic body and is then degraded (Figure 1) [5, 7]. Autophagosome formation is a fundamental process in autophagy that occurs in a variety of situations. Despite its importance, the molecular mechanism of autophagosome formation is still largely unknown. There are unsolved problems including the source of the autophagosome membrane, mechanism of membrane expansion, determinant of membrane curvature, and differentiation of the inner and outer membranes.

## 2. Cooperation of Proteins and Lipid Molecules in Autophagosome Formation

Currently, more than 30 genes involved in autophagy have been isolated in yeast and termed *ATG* (*a*u*t*opha*g*y-related) genes [9–11]. Among these genes, 18 *ATG* genes are essential for autophagosome formation and have known homologs in mammals, suggesting that the fundamental mechanism of autophagosome formation is conserved in eukaryotes. These 18 Atg proteins can be classified into 5 groups according to their functions (Figure 1) (for details, please refer to [12, 13]). These Atg proteins should effectively cooperate with lipid molecules to construct the autophagosome. Atg9 is the sole membrane-spanning protein among the 18 Atg proteins that are essential for autophagosome formation [14] and thus is one of the key molecules that links proteins and lipid molecules. Atg8 is also a key molecule in protein-lipid cooperation since this protein is covalently conjugated with phosphatidylethanolamine and localizes to the autophagosome [15–18]. The phosphatidylinositol (PtdIns) 3-kinase complex is another essential factor that mediates protein-lipid cooperation during autophagosome formation and will be further discussed in this paper.

## 3. PtdIns 3-Kinase in Autophagy

The budding yeast has only one PtdIns 3-kinase, Vps34, that is a class III kinase (hereafter, PtdIns 3-kinase indicates the class III PtdIns 3-kinase) that specifically phosphorylates PtdIns at the D-3 position of the inositol ring [19, 20]. *VPS34* was originally isolated in a genetic screen of mutants that erroneously secrete a soluble vacuolar protein CPY to the cell surface [21]. Thus, Vps34 is required for vacuolar protein sorting *via* the endosome. Later, *vps34*Δ cells were shown to be defective in autophagy, indicating that PtdIns 3-kinase is also involved in autophagy [22]. In *vps34*Δ cells, the autophagosome is not formed at all. The lipid kinase activity of Vps34 is essential for autophagosome formation as introducing a lipid kinase-dead form of Vps34 does not restore the autophagic activity of *vps34*Δ cells [23]. Vps15, Vps30/Atg6, and Atg14, which form a complex with Vps34 (see below), are also essential for autophagy [22, 24].

 PtdIns 3-kinase is also involved in autophagy in mammals. The addition of PtdIns(3)*P*, the enzymatic product of PtdIns 3-kinase, but not other phosphoinositides, enhances autophagic degradation in HT-29 cells [25]. PtdIns 3-kinase inhibitors, such as wortmannin and 3-methyladenine, suppress autophagy in mammalian cells. Similarly, knocking down the mammalian homolog of Atg14 [26–29] decreases autophagic activity. Thus, the role of PtdIns 3-kinase complex in autophagy is conserved in eukaryotes.

 In this paper, the current knowledge on how PtdIns 3-kinase functions in autophagy is summarized from the viewpoints of (i) assortment of PtdIns 3-kinase complexes into specific functions, (ii) dynamics of PtdIns(3)*P* during autophagy, and (iii) function of PtdIns(3)*P* in autophagy. Because yeast was used to make seminal advances on the role of PtdIns 3-kinase in autophagy, we focus on yeast studies and then briefly summarize recent results on mammalian autophagy.

## 4. Assortment of the PtdIns 3-Kinase Complex I for Autophagy

**Figure 2:**
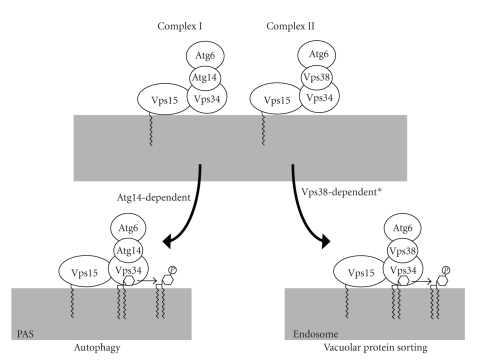
Mechanism by which the PtdIns 3-kinsase complexes are sorted into distinct processes. Vps34, a catalytic subunit, forms two distinct PtdIns 3-kinase complexes (complexes I and II). Complex I functions in autophagy, while complex II functions in the vacuolar protein sorting pathway. Atg14 is specifically integrated into complex I, while complex II contains Vps38 as a unique subunit. These specific subunits regulate the intracellular localization of these complexes, thereby determining their specific functions. Complex I localizes to the PAS in an Atg14-dependent manner, while complex II localizes to the endosome. *The endosomal localization of the complete complex II including Vps30/Atg6 depends on Vps38. However, Vps34 and Vps15 can localize to the endosome even in the absence of Vps38.

The sole PtdIns 3-kinase, Vps34, can participate in the two different biological processes, that is, vacuolar protein sorting and autophagy, due to the presence of two distinct PtdIns 3-kinase complexes (complexes I and II) [22]. Complex I functions in autophagy, while complex II is involved in vacuolar protein sorting (Figure 2). Both complexes exhibit PtdIns 3-kinase activity and share three common subunits, Vps15, Vps34, and Vps30/Atg6. Vps15 is a serine/threonine protein kinase that phosphorylates Vps34. Vps15-mediated phosphorylation of Vps34 is required to form the PtdIns 3-kinase complexes [30]. On the other hand, the lipid kinase activity of Vps34 is not required for complex formation. Vps15 tethers the PtdIns 3-kinase complexes to the membrane; although this protein is myristoylated at its N-terminus, the membrane association of the complex does not solely depend on myristoylation [31]. The function of Vps30/Atg6 within the PtdIns 3-kinase complexes is not well understood. However, Beclin 1, a mammalian homolog of Vps30/Atg6, interacts with various proteins involved in other biological processes, such as apoptosis, and is proposed to regulate the balance between autophagy and other biological processes [32]. A structural analysis and comparison of Vps30/Atg6 and Beclin 1 would be valuable to understand the function of Vps30/Atg6 and Beclin 1. In addition to common subunits, each complex also contains a unique factor. Atg14 is integrated into complex I, while Vps38 is specific to complex II. These additional subunits act as connecter molecules that bridge Vps30/Atg6 and Vps34 to allow complex formation [22]. In addition, these specific subunits play an essential role in determining the function of PtdIns 3-kinase complexes. Disruption of *ATG14* does not affect vacuolar protein sorting, while deletion of *VPS38* does not affect autophagy [22, 24]. Overexpression of Vps38 does not restore autophagic activity in *atg14*Δ cells. This strict assortment of the two complexes is conferred by the distinct intracellular localization of the two complexes [33]. Both complexes localize to the vacuolar membranes. In addition, each complex is targeted to a distinct compartment. Complex I is targeted to the pre-autophagosomal structure (PAS), a perivacuolar structure where Atg proteins assemble [34], while complex II is localized to the endosome. The localization of complex I to the PAS depends on Atg14 but not on Vps38 [33]. Whether the endosomal localization of complex II depends on Vps38 is not simple. Although Vps34 and Vps15 still localize to the endosome in *vps38*Δ cells, Vps30/Atg6 is no longer targeted to the endosome but is dispersed in the cytoplasm in *vps38*Δ cells. Thus, Vps38 is required to localize the entire complex II, including Vps30/Atg6, to the endosome, whereas the localization of Vps34 and Vps15 is Vps38 independent. The localization of complex II is not affected in *atg14*Δ cells. In summary, PtdIns 3-kinase complex I is sorted to function specifically in autophagy by localizing to the PAS depending on the specific Atg14 subunit. The PAS localization of Atg14 depends on Atg17 (a scaffold coiled-coil protein for Atg protein assembly), Atg9, and Atg13 [35]. Atg14 might regulate the function of complex I during autophagy by interacting with other Atg proteins at the PAS.

## 5. Dynamics of PtdIns(3)*P* during Autophagy

**Figure 3:**
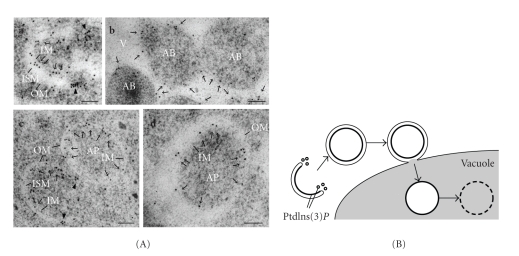
Dynamics of PtdIns(3)*P* during autophagy. (A) BJ2168 cells expressing mRFP-2xFYVE were cultured in nitrogen-depleted medium for 3.5 h and then subjected to immunoelectron microscopy with an affinity-purified anti-FYVE antibody. Gold particles on the inner membrane (IM) of the isolation membrane (ISM in (a) and (c)), the autophagosome (AP in (c) and (d)), and the autophagic body (AB in (b)) membranes are indicated by arrows. Arrowheads in (a) and (c) indicate gold particles near the tips of the isolation membranes. Ribosomes can be distinguished because they appear hazier than the gold particles. OM, outer membrane. V, vacuole. Bars, 100 nm. (B) Summary of the PtdIns(3)*P* dynamics during autophagy. PtdIns(3)*P*-enriched sites are shown in bold lines. Reproduced from [23] (Copyright 2008 by the Molecular Biology Society of Japan/Blackwell Publishing Ltd) and from [37] (Copyright 2008 by Landes Bioscience).

As mentioned above, the PtdIns 3-kinase activity of Vps34 is essential for autophagy. The enzymatic product of Vps34, PtdIns(3)*P*, can be monitored in yeast using the 2xFYVE domain, which specifically binds to PtdIns(3)*P in vitro* and *in vivo* [36]. In the logarithmic growth phase, PtdIns(3)*P* is detected on the late endosome and the vacuolar membrane [23], which is consistent with the localization of the PtdIns 3-kinase complexes. PtdIns(3)*P* is rarely detected at the PAS during logarithmic growth, suggesting that PtdIns(3)*P* is produced at a very low level or is transiently located at the PAS under conditions that do not induce autophagy. Upon autophagy induction, PtdIns(3)*P* is massively transported into the lumen of the vacuole. The transport of PtdIns(3)*P* depends on autophagosome formation. Fluorescence and electron microscopic analyses revealed that PtdIns(3)*P* is transported into the vacuole as a component of the elongating isolation membrane, autophagosome, and autophagic body, rather than as enclosed cargo [23]. The inner (concave) surface of the isolation membrane and autophagosome is enriched with PtdIns(3)*P*, while the outer (convex) surface does not appear to be rich in PtdIns(3)*P* (Figure 3). How PtdIns(3)*P* is specifically enriched on the inner surface is currently unknown. PtdIns(3)*P* is also detected on some amorphous membranes near the elongating tips of the isolation membrane. It is speculated that PtdIns(3)*P* near the elongating tips may be involved in constructing or maintaining the edges of the isolation membrane [37].

## 6. Function of PtdIns(3)*P* in Autophagosome Formation

Phosphoinositides often serve as a landmark on the membrane for phosphoinositide-binding proteins, thereby locally concentrating the effectors to allow subsequent processes to proceed. PtdIns(3)*P*-binding proteins often contain FYVE or PX domains through which they bind to PtdIns(3)*P* [36, 38, 39]. Among the Atg proteins, Atg20 and Atg24 contain a PX domain and are known to bind to PtdIns(3)*P* [40]. These proteins are not essential for autophagosome formation, although they are required for the Cvt (*c*ytoplasm to *v*acuole *t*argeting) pathway, a selective autophagy that occurs under conditions that do not induce autophagy [41, 42]. To date, there are no reports of an Atg protein with a typical FYVE domain.

 Atg18 and Atg21 are paralogous genes that bind to phosphoinositides, including PtdIns(3)*P*, although they do not contain FYVE or PX domains [43–45]. Atg18, but not Atg21, is essential for autophagosome formation, although autophagic activity is decreased in *atg21*Δ cells [46]. Atg18 is also essential to maintain the vacuolar morphology [44]. These dual functions of Atg18 are conferred by its ability to bind to both PtdIns(3,5)*P*2 and PtdIns(3)*P* at a putative phosphoinositide-binding motif, the FRRG sequence. Introducing mutations into this motif (e.g., substitution to FTTG) abolishes its ability to bind to PtdIns(3,5)*P*2 and PtdIns(3)*P in vitro* and causes abnormal vacuolar morphology and a significant reduction in autophagic activity *in vivo* [43–45]. Recent studies clarified that Atg18 must bind to PtdIns(3)*P* for autophagosome formation, while PtdIns(3,5)*P*2-binding is essential to maintain the vacuolar morphology [47, 48]. Atg18 forms a protein complex with Atg2. The formation of this complex does not require PtdIns(3)*P* and occurs even under conditions that do not induce autophagy. The interaction between PtdIns(3)*P* and Atg18 through the FRRG motif is required to efficiently recruit the Atg18-Atg2 complex to autophagic membranes (based on fluorescence microscopic analyses, it is difficult to clearly distinguish the PAS, isolation membrane, and autophagosome. Thus, in this paper, these structures will be referred to as autophagic membranes.) [48]. Thus, one of the essential roles of PtdIns(3)*P* in autophagy is to recruit the Atg18-Atg2 complex to autophagic membranes through the FRRG motif of Atg18. The residual autophagic activity detected in cells expressing the Atg18 (FTTG) variant is purportedly attributed to the function of the FRRG motif in Atg21 [49]. In addition to the PtdIns(3)*P*-Atg18 association, the interaction between Atg2 and a protein or lipid on autophagic membranes may synergistically and efficiently recruit the Atg18-Atg2 complex. Based on a comprehensive analysis of the hierarchy of Atg proteins at the PAS, it appears that the Atg18-Atg2 complex is directly involved in membrane formation rather than in early signaling events [35]. However, the precise function of the Atg18-Atg2 complex in autophagosome formation is still unknown. 

 In addition to recruiting the Atg18-Atg2 complex, PtdIns(3)*P* may have additional roles in autophagosome formation. The PAS localization of Atg8 and the Atg12-Atg5 conjugate associated with Atg16 (hereafter, Atg12 complex) is affected in *atg14*Δ cells [35]. Thus, these findings suggest that PtdIns(3)*P* is also involved, directly or indirectly, in efficiently recruiting Atg8 and the Atg12 complex to autophagic membranes. The Atg18-Atg2 complex does not affect the localization of the Atg12 complex and *vice versa* [35]. Thus, PtdIns(3)*P* likely independently recruits the Atg18-Atg2 complex and the Atg12 complex to autophagic membranes. The localization of Atg8 to membranes depends on conjugation with phosphatidylethanolamine [16], which is facilitated by the Atg12 complex [50]. It is possible that the inefficient recruitment of Atg8 in *atg14*Δ cells is due to the altered localization of the Atg12 complex. 

 In addition to these roles, it is possible that PtdIns(3)*P* itself directly affects the membrane architecture without the assistance of additional proteins. However, additional analyses, especially those using an *in vitro* system, are required to examine this possibility.

## 7. Involvement of PtdIns 3-Kinase in Regulating the Autophagic Activity and Size of the Autophagosome

As mentioned above, PtdIns(3)*P* serves as a landmark for the downstream Atg proteins that are thought to be directly involved in autophagosome formation. By modulating the amount of these downstream molecules at the site of autophagosome formation, PtdIns 3-kinase complex I may potentially influence the autophagic activity. Indeed, mild overexpression of Atg14 increases the autophagic activity [33]. 

 In contrast, truncating the C-terminal half of Atg14 causes a reduction in autophagic activity. Interestingly, the reduction in the autophagic activity in Atg14-truncated cells is mainly caused by the formation of smaller autophagosomes, suggesting that PtdIns(3)*P* regulates autophagosome size [33]. One possible explanation is that PtdIns(3)*P* directly affects the curvature of the isolation membrane, thereby determining the size of the autophagosome. An alternative possibility is that the formation of smaller autophagosomes in Atg14-truncated cells is due to the reduced recruitment of the downstream molecules that regulate the size of the autophagosome. Smaller autophagosomes are also formed in cells expressing Atg8 variants that have a defect in membrane tethering, one of the important functions of Atg8 [51]. Likewise, lower levels of Atg8 lead to a significant reduction in the size of the autophagosome [52]. Given that the localization of Atg8 is affected in *atg14*Δ cells, the effect of truncating Atg14 on autophagosome size might be attributed to the altered recruitment of Atg8.

## 8. PtdIns 3-Kinase and Autophagy in Mammalian Cells

In this paper, we introduced current knowledge on the function of PtdIns 3-kinase and PtdIns(3)*P* in autophagy, particularly in yeast. In this paragraph, we briefly summarize the relationship between PtdIns 3-kinase and autophagy in mammalian cells (for details, please refer to [53, 54]). 

 Based on pharmacological analyses and knockdown experiments of the subunits of the mammalian PtdIns 3-kinase complex, it is clear that PtdIns 3-kinase is involved in autophagy in mammalian cells [25, 26]. PtdIns 3-kinase is also required to sort lysosomal proteins *via* the endosome [55, 56]. Thus, the dual roles of PtdIns 3-kinase, that is, autophagy and sorting proteins to the lytic compartment *via* the endosome, are conserved from yeast to mammals. The recent identification of mammalian homologs of Atg14 and Vps38 (Atg14L and UVRAG, resp.) revealed that at least two distinct PtdIns 3-kinase complexes exist in mammals, as in yeast [26–29]. Moreover, like yeast, mammalian cells have a similar mechanism that sorts these PtdIns 3-kinase complexes into distinct functions. One difference in this sorting mechanism between yeast and mammals is the localization of the autophagy-specific PtdIns 3-kinase complex. The PAS has not been identified in mammalian cells. Instead of the PAS, Atg14L directs the autophagy-specific PtdIns 3-kinase complex to an ER subdomain. The ER localization of Atg14L depends on a protein complex that includes FIP200 [57], a protein thought to function as a scaffold, like yeast Atg17 [58]. In mammalian cells, autophagosome formation is preceded by the formation of the omegasome, a specialized subdomain of the ER [59]. At least some population of the autophagosome is formed inside the omegasome, which can be visualized by GFP-tagged DFCP (double FYVE-containing protein) [59, 60]. Atg14L is required for the formation of the omegasome [57, 59, 61]. In addition, Atg14L is required to recruit the mammalian homologs of the Atg12 complex, Atg8 (LC3), and Atg18 (WIPI-1) to autophagic membranes [57], which is consistent with the yeast process. WIPI-1 and -2 are thought to be direct effectors of PtdIns(3)*P* in mammalian autophagy, and WIPI-2 is proposed to have a major role [62].

 Beclin 1, a mammalian homolog of Vps30/Atg6, interacts with many proteins other than the core subunits of the PtdIns 3-kinase complexes. Ambra1 is one Beclin 1-interacting protein. Ambra1 positively regulates autophagy and plays a crucial role in neural development [63]. Beclin 1 also interacts with Rubicon, an RUN domain-containing protein, which is suggested to negatively regulate autophagosome maturation [28, 29]. Thus, autophagic activity is regulated, at least in some situations, through the interactions between Beclin 1 and regulatory proteins. Beclin 1 also interacts with Bcl2, an antiapoptotic protein, and is thought to regulate the balance between autophagy and apoptosis [32]. Based on these functions, Beclin 1 is thought to serve as a platform upon which multiple signals converge and regulate the crosstalk between autophagy and other processes [32]. This function of regulating the balance of multiple biological processes has not been reported for yeast Vps30/Atg6. Thus, compared to yeast Vps30/Atg6, Beclin 1 may have acquired additional roles during evolution. 

 Recent reports indicated that the balance between the production and turn over of PtdIns(3)*P* on autophagic membranes is important for the proper regulation of autophagy in mammals. Overexpression of PtdIns(3)*P* phosphatases decreases autophagic activity, whereas their knockdown or the expression of a dominant negative form enhances autophagic activity [64, 65]. Currently, it is unclear if this regulation by PtdIns(3)*P* phosphatases also occurs in yeast.

 Taken together, the overall functional framework of PtdIns 3-kinase in autophagy is conserved from yeast to mammals. The PtdIns 3-kinase complex has a specific function in autophagy that involves incorporating a specific subunit, Atg14. Atg14 directs the autophagy-specific PtdIns 3-kinase complex to the site of autophagosome formation depending on the scaffold proteins, including Atg17. PtdIns(3)*P* produced at the formation site serves as a landmark for downstream molecules such as Atg18. One difference between yeast and mammals is that mammalian Atg14 directs the PtdIns 3-kinase complex to the ER, while yeast Atg14 directs the complex to the PAS.

## 9. Autophagosome Formation as a Suitable Model to Study PtdIns 3-Kinase

Autophagy has ideal features that make it conducive to study the dynamics and function of PtdIns 3-kinase, PtdIns(3)*P*, and related effectors. First, autophagy can be easily induced and cancelled by depleting and replenishing nutrients, respectively. Autophagy is also effectively induced, particularly in yeast cells, by treating with rapamycin, an inhibitor of the TOR complex that negatively regulates autophagy under nutrient-rich conditions. This feature allows researchers to detect autophagic activity with low background and high sensitivity. Second, most of the yeast *atg* mutants exhibit almost no phenotype other than autophagy under nutrient-rich conditions, allowing us to maintain these mutants. Third, autophagosomes are much larger than secretory vesicles, endocytic vesicles, and the intraluminal vesicles of multivesicular bodies. Thus, we can easily distinguish the autophagosome from these vesicles and can monitor autophagosome formation using fluorescence microscopy, especially in mammalian cells. Fourth, there are several available markers for autophagic membranes. Atg8 (LC3 in mammalian cells) is the most established marker for the isolation membrane and the autophagosome. The Atg12 complex labels the elongating isolation membrane but not the completed autophagosome in mammalian cells. The FYVE domain clearly labels the isolation membrane, autophagosome, and autophagic body in yeast, and DFCP is a suitable marker for the omegasome in mammals. Based on these advantages, studies on PtdIns 3-kinase in autophagy may provide not only novel knowledge on the mechanism of autophagosome formation but also a general idea about the dynamics and function of phosphoinositides.

## 10. Future Research

Although the regulation and function of PtdIns 3-kinase in autophagy have been gradually unveiled, there are still some unsolved issues. The relationship between PtdIns 3-kinase complex formation and the enzymatic activity of Vps34 has not been revealed in detail. Lysates of *atg6*Δ cells and wild-type cells exhibit indistinguishable PtdIns 3-kinase activity [22]. Additional quantitative analyses using recombinant proteins of each subunit may clarify the potential involvement of Atg14, Vps38, and Vps30/Atg6 in modulating the enzymatic activity of Vps34. Another unsolved issue concerning the regulation of PtdIns 3-kinase is how Atg14 directs the autophagy-specific PtdIns 3-kinase complex to the PAS and the ER subdomain in yeast and mammals, respectively. In both organisms, the Atg17 complex is required for the proper localization of Atg14 [35, 57]. The N-terminal region of Atg14 contains conserved cysteine residues that are essential for targeting Atg14 to the ER in mammalian cells [61]. These reports will help solve the targeting mechanism of the autophagy-specific PtdIns 3-kinase complex.

 The mechanism by which PtdIns(3)*P* is specifically enriched on the isolation membrane is also an important issue. The PtdIns(3)*P*-rich amorphous membranes near the tips of the isolation membrane should also be characterized.

 Finally, the function of PtdIns(3)*P* and the downstream molecules should be studied in more detail. How the Atg18-Atg2 complex functions in autophagosome formation is still largely unknown. Furthermore, there may be additional unidentified effectors. In addition, the possible direct involvement of PtdIns(3)*P* in modulating the curvature of the isolation membrane without assistance from other effectors should be examined.
